# The prognostic value of a Methylome-based Malignancy Density Scoring System to predict recurrence risk in early-stage Lung Adenocarcinoma

**DOI:** 10.7150/thno.44229

**Published:** 2020-06-18

**Authors:** Lu Yang, Jing Zhang, Guangjian Yang, Haiyan Xu, Jing Lin, Lin Shao, Junling Li, Changyuan Guo, Yanru Du, Lei Guo, Xin Li, Han Han-Zhang, Chenyang Wang, Shannon Chuai, Junyi Ye, Qiaolin Kang, Hao Liu, Jianming Ying, Yan Wang

**Affiliations:** 1 Department of Medical Oncology, National Cancer Center/National Clinical Research Center for Cancer/Cancer Hospital, Chinese Academy of Medical Sciences and Peking Union Medical College, Beijing, 100021, China.; 2 Department of Pathology, National Cancer Center/National Clinical Research Center for Cancer/Cancer Hospital, Chinese Academy of Medical Sciences and Peking Union Medical College, Beijing, 100021, China.; 3 Department of Comprehensive Oncology, National Cancer Center/National Clinical Research Center for Cancer/Cancer Hospital, Chinese Academy of Medical Sciences and Peking Union Medical College, Beijing, 100021, China.; 4 Burning Rock Biotech, Guangzhou, 510300, China.

**Keywords:** stage IA lung adenocarcinoma, disease-free survival, methylome-based malignancy density, genomic and epigenetic signatures, recurrence

## Abstract

Current NCCN guidelines do not recommend the use of adjuvant chemotherapy for stage IA lung adenocarcinoma patients with R0 surgery. However, 25% to 40% of patients with stage IA disease experience recurrence. Stratifying patients according to the recurrence risk may tailor adjuvant therapy and surveillance imaging for those with a higher risk. However, prognostic markers are often identified by comparing high-risk and low-risk cases which might introduce bias due to the widespread interpatient heterogeneity. Here, we developed a scoring system quantifying the degree of field cancerization in adjacent normal tissues and revealed its association with disease-free survival (DFS).

**Methods:** We recruited a cohort of 44 patients with resected stage IA lung adenocarcinoma who did not receive adjuvant therapy. Both tumor and adjacent normal tissues were obtained from each patient and subjected to capture-based targeted genomic and epigenomic profiling. A novel methylome-based scoring system namely malignancy density ratio (MD ratio) was developed based on 39 patients by comparing tumor and corresponding adjacent normal tissues of each patient. A MD score was then obtained by Wald statistics. The correlations of MD ratio, MD score, and genomic features with clinical outcome were investigated.

**Results:** Patients with a high-risk MD ratio showed a significantly shorter postsurgical DFS compared with those with a low-risk MD ratio (HR=4.47, P=0.01). The MD ratio was not associated with T stage (P=1), tumor cell fraction (P=0.748) nor inflammatory status (p=0.548). Patients with a high-risk MD score also demonstrated an inferior DFS (HR=4.69, P=0.039). In addition, multivariate analysis revealed *EGFR* 19 del (HR=5.39, P=0.012) and MD score (HR= 7.90, P=0.01) were independent prognostic markers.

**Conclusion:** The novel methylome-based scoring system, developed by comparing the signatures between tumor and corresponding adjacent normal tissues of individual patients, largely minimizes the bias of interpatient heterogeneity and reveals a robust prognostic value in patients with resected lung adenocarcinoma.

## Introduction

Curative resection is the standard of care for patients with stage I non-small-cell lung cancer (NSCLC). Current NCCN guidelines do not recommend the use of adjuvant chemotherapy for stage IA lung adenocarcinoma patients with R0 surgery. However, approximately 20% to 40% of patients with stage IA NSCLC experience recurrence within 5 years after surgery 1-3. Effective recurrence prediction in this group of patients is needed. In addition, although surveillance imaging is also recommended for all resected patients, the level of supportive evidences is low and adherence rates are limited 4, 5. Hence, stratifying patients according to the predicted recurrence risk may tailor adjuvant therapy and surveillance imaging to patients with a higher risk 6. Tremendous efforts have been invested to identify clinical and molecular characteristics that might help predict recurrence risk in addition to TNM stage in resected lung cancers 7-11. Clinical parameters including but not limited to vascular invasion, visceral pleural involvement (VPI), poorly differentiated tumors, and wedge resections, as well as molecular signatures including Ki-67 expression, *MACC1* gene amplification, microRNA expression were found to be indicative of prognosis; however, the prediction performance was still not satisfactory 12, 13. In clinical practice, the prognostic features commonly used in clinical decision-making remain to be the tumor stage and the patient's performance status 14.

DNA methylation, a primary epigenetic modification in mammalian genome often occurring at CpG islands, is an important mechanism in gene and microRNA expression regulation 15 as well as in alternative gene splicing 16. It plays an essential role in the development as well as the progression and metastasis of lung cancer 17. Compared with mutation, copy number variation (CNV) and gene/microRNA expression 18-20, DNA methylation was utilized as the most promising marker for the early detection of cancer due to its stability and being easily detected qualitatively and quantitatively 21, 22. In the past decade, there has been a blossoming of studies on early detection and risk of recurrence of lung cancer by analyzing methylation signatures 23-26. However, limitations also exist such as small sample size, limited number of selected genes and qualitative instead of quantitative measurement of DNA methylation, which might explain the low reproducibility of these assays 25.

The concept of field cancerization (also unknown as field effect or field defect) was first introduced by Slaughter et al. in 1953 to describe a field of normal-appearing tissue that has been preconditioned by unknown processes so as to predispose it towards development of cancer 27. Even though initially based on histological observations, field cancerization now has been demonstrated to occur at the molecular level. Precancerous cells adjacent to the tumor cells acquiring tumor-primed genetic alterations have been identified in various organs 28-30. In addition, epigenetic field cancerization has also been discovered in various types of cancers, including lung 31, stomach 32, liver 33, colon 34, bladder 35 and so on. Field cancerization is now recognized to underlie the development of many types of cancer, including lung carcinomas 36, 37 and might have an etiologic role in a substantial number of recurrences 38. Therefore, sensitive detection of cancerized fields at high risk of developing malignancy by molecular profiling is highly desirable. However, the whole cancerization idea can't pinpoint the risk. Instead, biomarker based on quantifying the underlying evolutionary process within the cancerized field might have a more robust prognostic value 39. Genetic diversity, genomic instability and size of clonal expansion have been identified as such evolutionary markers showing prognostic value in blood, Barrett's esophagus and ulcerative colitis 40-43. Under the hypothesis that the field cancerization of adjacent tissues from the surgery site might be associated with patient's outcome, we developed a novel methylome-based scoring system namely malignancy density ratio (MD ratio) to characterize the degree of field cancerization of the adjacent normal tissues. The aim of the present study is to investigate the association of this MD ratio with the risk of disease recurrence in resected stage IA lung adenocarcinomas.

## Methods

### Patient information

We retrospectively recruited a total of 44 stage IA (T1a/T1b, N0) lung adenocarcinoma patients who underwent curative resection (without adjuvant therapies) from Cancer Hospital, Chinese Academy of Medical Sciences and Peking Union Medical College between October 2011 and November 2015 (follow-up through April 2018). Curative resection was defined as the removal of all malignant (cancerous) tissue to cure the disease. The histopathological and clinical characteristics of patients as well as disease-free survival (DFS) were collected. The study was approved by the institutional review board of Cancer Hospital, Chinese Academy of Medical Sciences and Peking Union Medical College. All patients provided written informed consent, in accordance with the Declaration of Helsinki.

### DNA isolation from tissues

Paired tumor tissues and adjacent normal tissues were obtained during surgery. Adjacent normal tissues were biopsied >5cm distant from resection margins for lobectomy and segmentectomy, and > 3cm from margins for wedge resection. The absence of tumor cells in the normal tissue samples was confirmed by histopathological assessment. Both samples were subjected to DNA isolation using the QIAamp DNA FFPE Tissue Kit (Qiagen, Valencia, CA, USA) according to the manufacturer's instructions. DNA was quantified with the Qubit 2.0 fluorimeter (ThermoFisher Scientific, Waltham, MA, USA).

### Bisulfite sequencing

Forty-four paired tumor and adjacent normal tissues were sequenced using a capture-based bisulfite sequencing panel as described previously 44. The bisulfite sequencing (BS-seq) library was prepared using the brELSATM method (Burning Rock Biotech, Guangzhou, China). Briefly, purified DNA was treated with sodium bisulfite (D5046, EZ-96 DNA Methylation-Lightning™ MagPrep, Zymo Research, Orange, CA, USA). Subsequently, the converted single-strand DNA molecules were ligated to a splinted adapter, and amplified by a uracil-tolerating DNA polymerase to generate whole-genome BS-seq libraries. Custom-designed methylation profiling RNA baits were used for target enrichment which covers 80,672 CpG sites and spans 1.05 mega base of human genome. The target libraries were subsequently quantified by real-time PCR (Kapa Biosciences Wilmington, MA, USA) and sequenced on NovaSeq 6000 (Illumina, San Diego, CA, USA) with an average sequencing depth of 1,000X.

### Methylation data analysis

Trimmomatic (v.0.32) was used to remove custom adaptor sequences and low-quality bases. Paired-end reads were aligned to C to T- and G to A-transformed hg19 genome by BWA-meth (v.0.2.2) 45. After alignment, duplicate reads were marked by samblaster (v.0.1.20) 46, and low mapping quality (MAPQ<20) or improper pairing reads were removed by sambamba (v.0.4.7) 47 from further downstream analyses. Paired reads were merged by clipping overlapping reads to avoid double-counting of methylation calls. Methylation blocks (MBs) were defined as the genomic region consisting of the neighboring CpG sites which were not only close on distance but also correlated on methylation level. A total of 8,312 MBs were generated from 80,672 CpG sites using a proposed region-defined algorithm. Within all MBs, 84% were annotated in genes with 59% in promoter regions, 7% in exons and 18% in introns. We defined M and U as the methylated and unmethylated reads aligned on a CpG site, respectively. M_ij_ and U_ij_ sum the M and the U in the jth MB for the ith patient, respectively (Table S1). We defined βji as the methylation signature in the jth MB of the ith patient following the formula: β_ij_ = M_ij_/(M_ij_+U_ij_).

### Generation of the MD ratio and MD score

MD ratio and MD score were generated as illustrated in Figure S1.The cancer-specific blocks namely differential methylated blocks (DMBs) were selected by testing the signature difference between tumor and normal tissues, and MBs with significant difference (P<0.05) were chosen. The personalized DMBs were selected by comparing the differential signatures in tumor and paired normal tissues of a given patient. We estimated the baseline methylation signature β_j_^(0)^ based on the methylation data of normal lung tissues from an internal database via maximum likelihood estimation (MLE). We defined β_ij_^(a)^ and β_ij_^(t)^ as the methylation signature for adjacent normal tissue and tumor tissue of the ith patient respectively, which follow the equation:


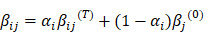


Assuming that the methylation signature follows a mixed beta-binomial model, the α_i_ (MD ratio) of each patient was estimated by MLE which reflects the proportion of methylation signature in the adjacent normal tissue shared by its corresponding malignant tumor tissue. Hessian Matrix was used to estimate the variance of the MD ratio estimator. MD score was obtained by Wald statistics under the null hypothesis of MD ratio equals to 0, representing the density of malignant signature present in the adjacent normal tissue of an individual patient.

### Targeted DNA sequencing for genomic characterization

Capture-based targeted sequencing for somatic mutation profiling was performed on 44 tumor samples using a panel consisting of 520 cancer-related genes (Table S2). Ten randomly selected adjacent normal tissues were also subjected to capture-based targeted sequencing for mutation profiling. The NGS library was prepared as previously described 48 and sequenced on a NextSeq 500 (Illumina, Inc., San Diego, CA, USA) with pair-end reads with an average depth of 1,000X. The sequencing data in a FASTQ format were mapped to the human genome (hg19) using BWA aligner 0.7^49^. Local alignment optimization, mark duplication, and variant calling were performed using the Genome Analysis ToolKit (GATK) 3.2^50^, Picard (http://picard.sourceforge. net/) and VarScan^51^. Gene translocations were identified with FACTERA 52 and the CNV was called with an in-house algorithm based on sequencing depth 53.

### Statistical analysis

Statistical analysis was performed using R version 3.3.3 software. Differences in groups were calculated and presented either by Fisher's exact test or paired two-tailed Student's t-tests, as appropriate. Wilcox test was used to study the correlation of MD ratio with tumor cell fraction in tumor tissue. A receiver operating characteristic (ROC) curve was generated to identify the cut-off of MD ratio. Kaplan-Meier analysis was used to estimate survival functions, and a log-rank test was used to determine the difference in the survival curves between groups. p < 0.05 was considered statistically significant. Possible predictors of DFS were investigated using Cox univariate or multivariate proportional-hazards analysis.

## Results

### Characteristics of patients

We retrospectively recruited 44 patients with stage IA lung adenocarcinoma who underwent curative resection without adjuvant therapy. The demographic and clinical characteristics of patients were summarized in Table 1. The median age of this cohort was 61 years, ranged from 40 to 81 years. Among them, 28 (63.6%) were males. Twenty-five (56.8%) patients had a history of smoking. The median tumor diameter was 1.5cm. The perineural invasion (PNI) and spread through air spaces (STAS) were present in 6 (13.6%) and 18 (40.9%) patients, respectively. Visceral pleural invasion (VPI) was not found in any of the patients. Eleven (25%) patients underwent pulmonary lobectomy and 31 (70.5%) had thoracoscopic lobectomy. Only 2 (4.5%) were treated with thoracoscopic wedge resection. Twenty-nine (65.9%) patients had right lung resected. After a median follow-up time of 41.5 months, the median DFS of this cohort was 33 months, ranged from 3.3 - 69.3 months. None of the characteristics significantly correlated with DFS (Table 1).

### The prognostic values of MD ratio and MD score

**Figure 1:**
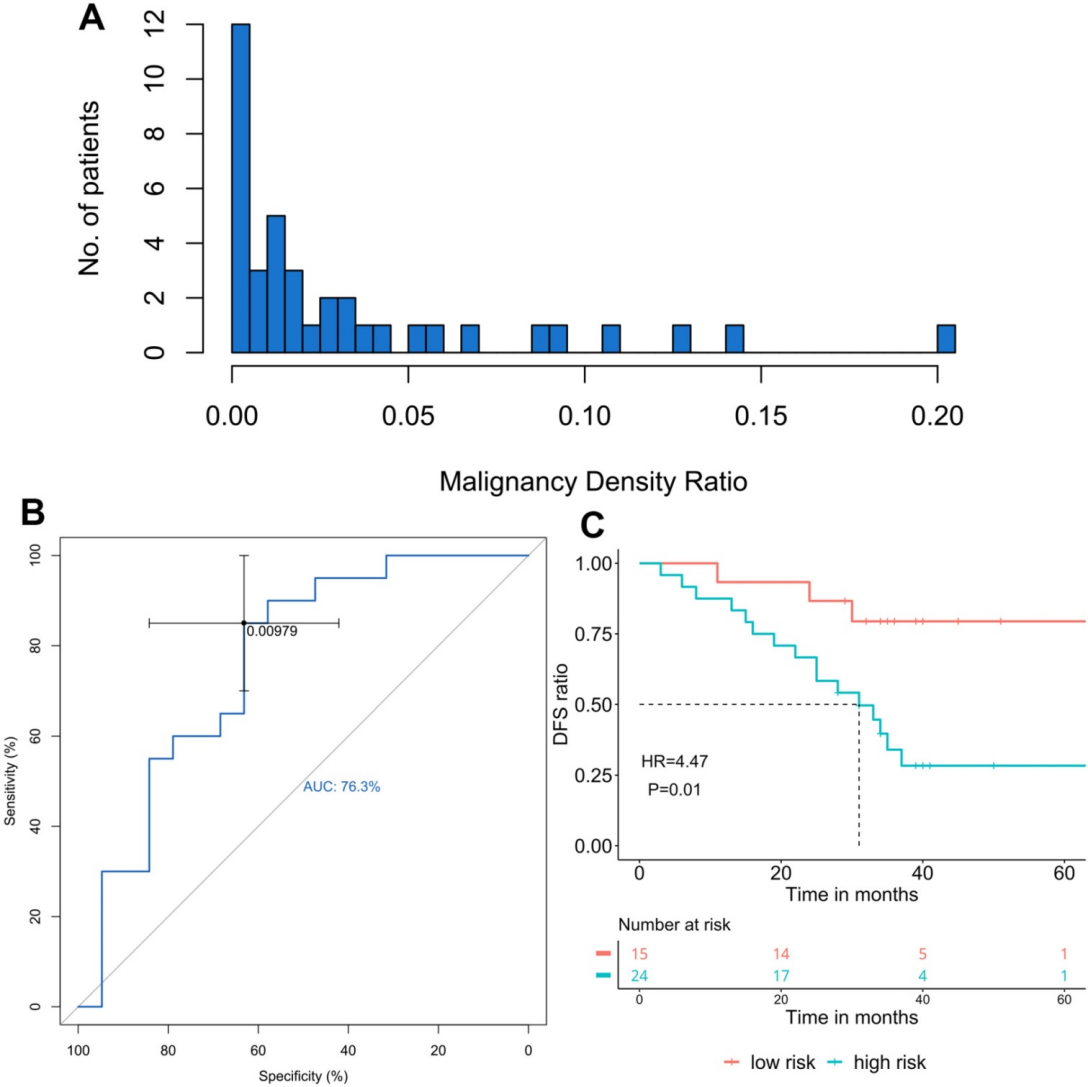
The association of malignancy density (MD) ratio and prognosis in resected stage IA adenocarcinoma patients (n=39) A. The distribution of MD ratio; B. The ROC curve of MD ratio to discriminate patients relapsed from those did not during the follow-up; C. The disease-free survival (DFS) in patients with different MD ratios, cut-off=0.00979.

Paired tumor and adjacent normal tissue samples from 44 patients were subjected to bisulfite sequencing and 39 of them generated data with sufficient quality for both paired samples, therefore, underwent further methylation analysis and MD ratio calculation. The cancer-specific blocks for each patient were selected by comparing the methylome-based signatures between the patient's tumor and adjacent normal tissues. MD ratio of each patient was estimated by MLE, which reflects the proportion of malignant methylation signal in the adjacent normal tissue shared by its corresponding malignant tumor tissue. MD ratio ranged from 0 to 0.2 with a majority of samples close to 0 (Figure 1A). Next, we performed a ROC analysis to derive a cut-off for MD ratio to discriminate patients who relapsed during the follow-up from those who did not (Figure 1B). MD ratio of 0.00979 was identified, with an area under curve of 76.3%. We observed a significantly shorter postsurgical DFS in patients with a MD ratio greater than 0.00979 (high-risk) compared with those with a low-risk MD ratio (33 months vs. NR, HR=4.47, P=0.01, Figure 1C).

We also investigated the correlation between the MD ratio and some histological characteristics, including T stage, tumor cell fraction and inflammatory status (Table 2). No significant correlation between MD ratio and T1a/T1b was observed (P=1). Furthermore, neither tumor cell fraction (P=0.548) nor inflammatory status (P=0.748) of adjacent normal tissue correlated with MD ratio, suggesting that the MD ratio derived from this panel is an independent predictive factor for prognosis. Furthermore, we investigated the associations of tumor cell fraction and inflammatory status with DFS by univariate analysis, and neither of them significantly correlated with DFS (HR=0.125, P=0.097; HR=0.747, P=0.53).

**Figure 2:**
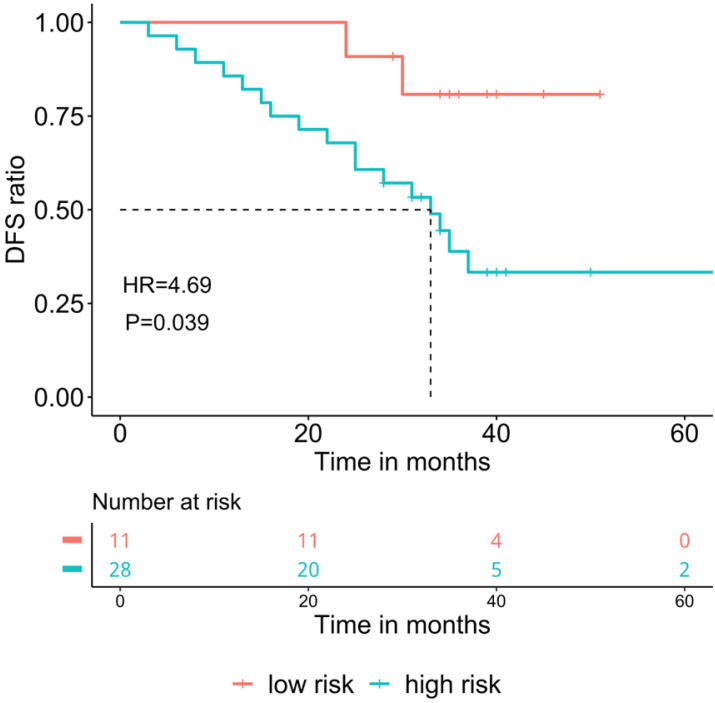
The association of malignancy density (MD) score and prognosis in resected stage IA adenocarcinoma patients (n=39). The cut-off for high-risk group was 1.96, DFS: Disease-free survival.

Since MD ratio is a point-estimation on the proportion of tumor-shared methylation signature in the adjacent normal tissue, a raw statistic which lacks the statistical significance. We further developed an MD score, which was obtained by Wald statistics under the null hypothesis that MD ratio equals to 0. Statistically, the MD score is positively correlated with the probability (p-value) of the condition that the malignant signature presents in the adjacent normal tissue of a given patient. As a Wald statistic, MD score follows a chi-square distribution with 1 degree of freedom and takes the variance of MD ratio into consideration, so it is a more robust marker to stratify patients. We used 1.96 (p-value<0.05) as the calling threshold of the high-risk score. Patients with a high-risk MD score also showed significantly poorer prognosis (DFS: 33 months vs. NR, HR=4.69, P=0.039, Figure 2).

### The genomic profile and correlation with prognosis

**Figure 3:**
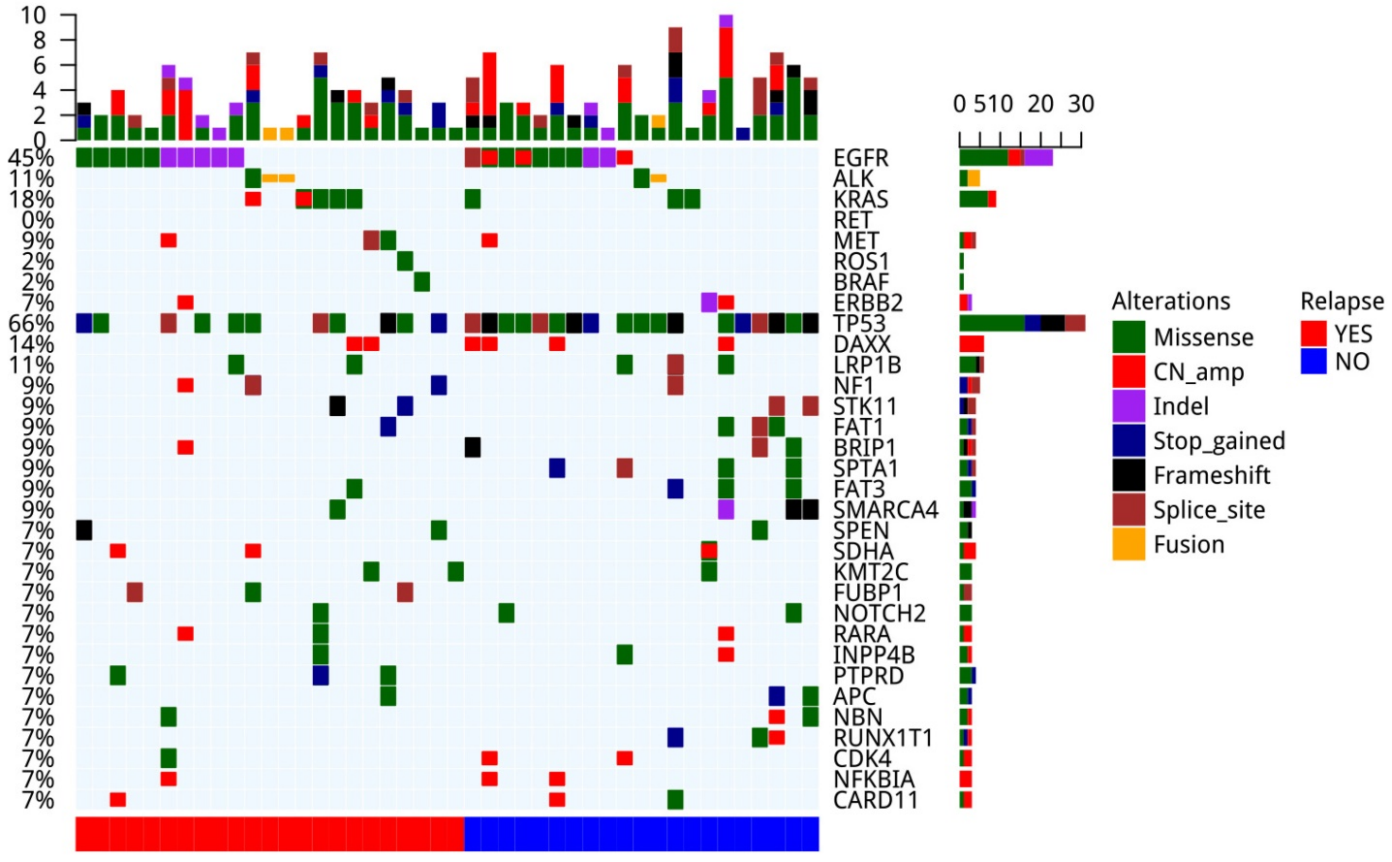
The landscape of genomic mutations in tumor lesions of patients (n=44).

We performed a comprehensive analysis on the genomic alternations in 44 tumor tissue samples (Figure 3). Driver mutations were detected in 33 out of the 44 samples (70%), including *EGFR* driver mutations (n=18, 41%), *ALK* fusion (n=3, 7%), *KRAS* G12V (n=7, 16%), *MET* amp (n=2, 5%), *MET* 14 splicing (n=1, 2%), *ERBB2* amp (n=2, 5%) and* ERBB2* 20ins (n=1, 2%). The most common concomitant alternations occurred in *TP53* gene (66%), followed by *DAXX* (14%) and *LRP1B* (11%). *TP53* (P=0.011) and *SPTA1* (P=0.044) mutated less frequently in patients with recurrence compared with those without (Figure S2).

**Figure 4:**
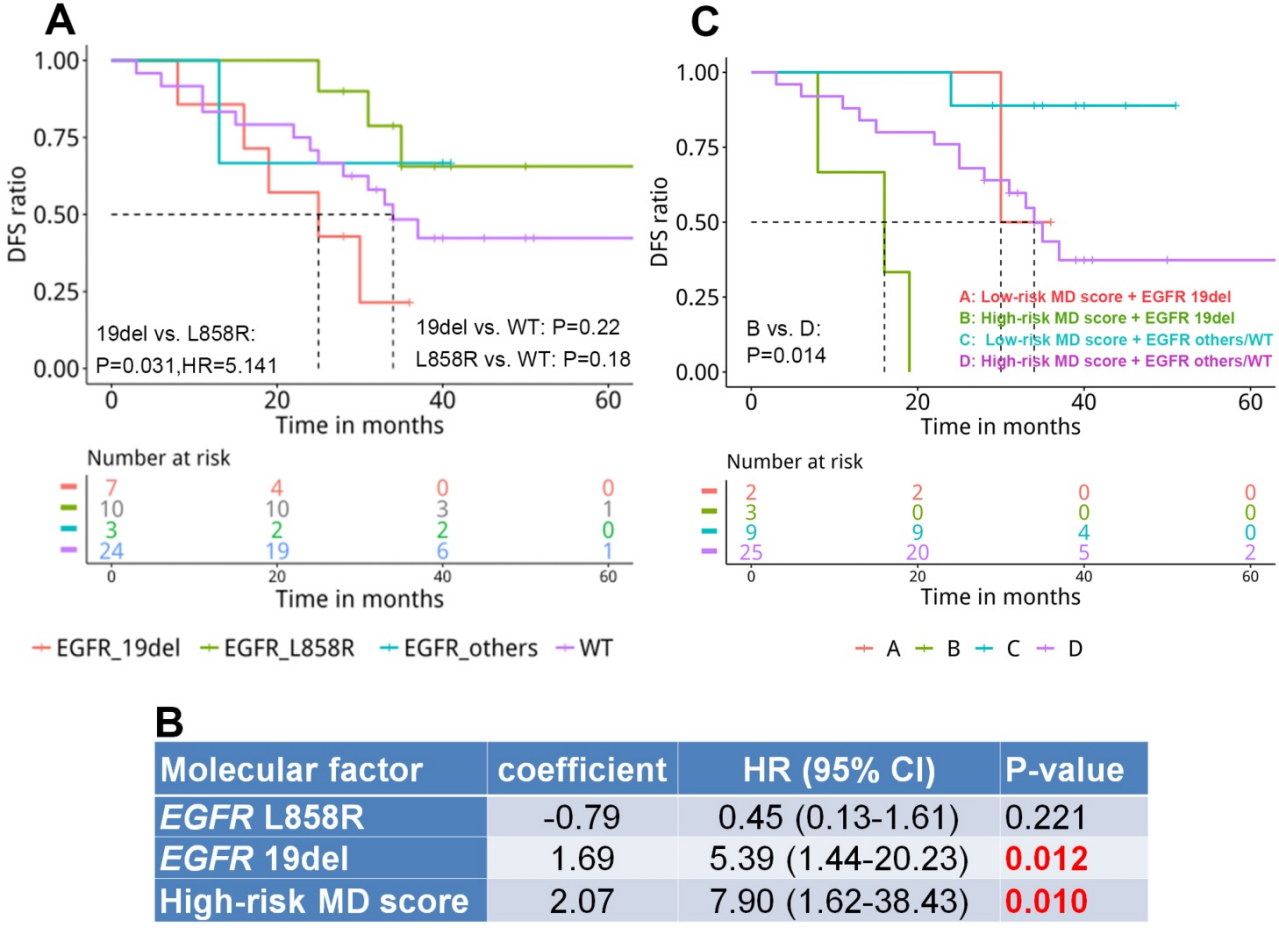
The prognostic value of genomic and epigenetic signatures in resected stage IA adenocarcinoma patients. A. The correlation of *EGFR* driver mutation subtype with disease-free survival (DFS) (n=44); B. Cox multivariate proportional-hazards analysis of the correlation of molecular factors with disease-free survival (n=39); C. The correlation of *EGFR* driver mutation subtype and MD score with DFS (n=39).

By investigating the correlation between mutation landscape and DFS, we revealed that patients harboring *EGFR* 19del displayed significantly shorter DFS compared with those carrying *EGFR* L858R (25 months vs. NR, HR=5.141, P=0.031, Figure 4A). A median DFS of 34 months was observed in WT patients without significant difference compared with *EGFR*-mutant groups.

We randomly selected 10 adjacent normal tissues for somatic mutation profiling. All sample had no mutation identified except for one, which was detected with an *EGFR* G719A with an allele frequency (AF) of 1.43%. This patient's paired tumor tissue also harbored *EGFR* G719A with a higher AF of 40.30%. Concordantly, both MD ratio and MD score classified the patient into high-risk group. On the other hand, 7 out of the 9 patients, whose adjacent normal tissues were free of mutation, were stratified into to high-risk by both MD ratio and score, suggesting a superior sensitivity of MD ratio/score to predict recurrence over somatic mutation.

### Stratifying patients with both genomic signature and MD score

MD score and* EGFR* driver mutation, that were significantly associated with DFS in univariate analysis, were included in Cox multivariate proportional-hazards analysis. The multivariate analysis revealed that *EGFR* 19 del (HR=5.39, P=0.012) and MD score (HR= 7.90, P=0.01) remained as predictors for the risk of developing postsurgical recurrence (Figure 4B). Next, we stratified patients by integrating both prognostic factors and assessed the difference in DFS among subgroups. Our results displayed a significantly shorter DFS in patients with a high-risk MD score and an *EGFR* 19 del compared with those with a high-risk MD score but without the *EGFR* 19 del (*EGFR* others or WT)(P=0.014, Figure 4C). No significant difference between other subgroups was observed due to the small number of patients.

## Discussion

Since the global change of DNA methylation occurs very early in the process of carcinogenesis, DNA methylation has been considered as one of the most powerful biomarkers for early detection and screening in cancer. Extensive efforts have been invested in the discovery of methylation biomarkers for lung cancer screening and early detection 25, 54-56; however, fewer studies have assessed the utility of aberrant methylation profiles to predict recurrence risk after resecting NSCLC. Brock et al. demonstrated that methylation of the promoter region of *p16*, *CDH13*, *RASSF1A*, and *APC* was associated with early recurrence in surgically-treated patients with stage I (T1-2N0) NSCLC 23. Belinsky et al. reported the methylation detection of 8 selected genes (*CDKN2, MGMT, DAPK1, RASSF1, GATA4, GATA5, PAX5α* and *PAX5β*) in sputum and blood had prognostic value for recurrence in stage IA (pT1N0) or stage IB (pT2N0) NSCLC 24. More recently, Wang and colleagues derived a classifier based on 16-CpG sites to predict the overall survival of lung adenocarcinoma patients 26. Sandoval et al. discovered a methylation signature based on 10 sites (*HOXA9, C1orf114, TRH, HIST1H4F, SP9, PCDHGB6, OTX2, NPBWR1, TRIM58*, and* ALX1)* that effectively distinguished stage I NSCLC patients with high recurrence risk and low risk 57.Conventionally, molecular or epigenetic biomarkers are often identified by comparing the genomic or epigenomic landscapes of two groups with diverse outcomes. This identification method might be influenced by selection bias present in the groups due to the widespread interpatient and intra-patient heterogeneity 6. Notably, our panel covers 7 of the 10 sites identified in Sandoval et al. 2013 57 (Table S3). However, neither any single nor combination of the 7 sites correlated significantly with DFS in our cohort (Table S3, Figure S3).

We established a scoring system by comparing the methylation signatures in a tumor and its adjacent normal tissue of the same individual to reflect the malignant progression of a cancerized field. This classification is independent of patients' DFS and largely attenuates the effect of interpatient heterogeneity. MD ratio demonstrated a significant association with DFS, which was independent of clinicopathological factors including T stage and tumor cell fraction, suggesting its robustness in a heterogeneous population. In addition, epigenetic modifications arising from exposure to the environment, such as the disturbance of DNA methylation in the context of transcriptional level, as well as the signal-transduction and cellular pathways of the inflammatory cascade, has been closely linked with the pathophysiology of inflammatory diseases 58, 59. Conceivably, the inflammatory status might affect the methylation signatures in the tissue. In our study, the methylome-based MD ratio was independent of the inflammatory status (congestion, emphysema, pulmonary bullae with interstitial fibrosis or obstructive pneumonia) in the adjacent normal tissue because our scoring system was based on the degree of field cancerization of the adjacent normal tissues. In other words, it is a comparison between the tumor tissue and adjacent normal tissue of the same individual and minimizes the selection bias that could be introduced during the identification of conventional markers. Our method indicates the merit of evolutionary markers over selective markers.

The prognostic role of* EGFR* mutations also remains controversial in patients with resectable lung adenocarcinoma 60, 61. A recent study 62 conducted in 835 patients, who underwent complete surgical resection for lung adenocarcinoma without EGFR TKIs as a neoadjuvant or adjuvant therapy, showed that patients with 19del had a significantly higher incidence of extrathoracic recurrence than patients with L858R (p =0.004), and the L858R group had a significantly longer recurrence-free survival than the WT group (p < 0.001) and the 19del group (p = 0.016). Concordantly, our results also showed that 19del was an independent genetic predictor significantly associated with a worse prognosis.

Our study was limited by the number of patients enrolled and its retrospective and non-randomized nature. To extend the interesting findings from our work, prospective studies with larger cohorts are required to validate the prognostic value of the MD ratio/score. Nonetheless, we established a methylome-base scoring system to quantitatively assess the malignant progression of adjacent normal tissue based on personalized cancer-specific methylation signatures of individual. The scoring system revealed robust prognostic value in patients with resected stage IA lung adenocarcinoma and was independent of clinicopathological factors and genetic signatures. Using it to characterize the risk of lung cancer and recurrence will facilitate a personalized utility of adjuvant therapy and surveillance imaging in completely resected NSCLCs.

## Supplementary Material

Supplementary figures and tables.

## Figures and Tables

**Table 1:** Clinicopathological characteristics of patients

Characteristics	All (n=44)	Correlation with DFS
**Age, years**		P=0.134
Median (Min, Max)	61 (40,82)	
**Gender, n (%)**		P=0.051
Female	16 (36.4%)	
Male	28 (63.6%)	
**Smoking history, n (%)**		P=0.151
No	18 (40.9%)	
Yes	25 (56.8%)	
Unknown	1 (2.3%)	
**Tumor diameter, cm**		P=0.068
Median (Min, Max)	1.5 (0.7,2.0)	
**PNI, n (%)**		P=0.512
No	38 (86.4%)	
Yes	6 (13.6%)	
**STAS, n (%)**		P=0.141
No	24 (54.6%)	
Yes	18 (40.9%)	
Unknown	2 (4.5%)	
**VPI, n (%)**		-
No	44 (100%)	
**Surgical procedure, n (%)**		P=0.085
Pulmonary lobectomy	11 (25%)	
Thoracoscopic lobectomy	31 (70.5%)	
Thoracoscopic wedge resection	2 (4.5%)	
**Surgery Location, n (%)**		P=0.06
Right lung	29 (65.9%)	
Left lung	15 (34.1%)	
**Follow-up time, months**		-
Median (Min, Max)	41.5 (8.1, 74.9)	
DFS, months		-
Median (Min, Max)	33 (3.3, 69.3)	

PNI: Perineural invasion; STAS: Spread through air spaces; VPI: Visceral pleural invasion; DFS: Disease-free survival. P-value was calculated by Cox univariate proportional-hazards analysis.

**Table 2:** The association of MD ratio with histopathological features

Characteristics		MD ratio		P-value
	All (n=39)	Low-risk (n=15)	High-risk (n=24)	
**Stage, no. (%)**				P^a^=1
T1a	4 (10.3%)	1(6.7%)	3 (12.5%)	
T1b	35 (89.7%)	14(93.3%)	21 (87.5%)	
**Adjacent normal tissue status, no. (%)**				P^a^=0.748
Normal	19(48.7%)	8(53.3%)	11 (45.8%)	
Inflammatory *	20(51.3%)	7(46.7%)	13 (54.2%)	
% tumor cells in tumor, median (range)	0.5(0.1-0.9)	0.5(0.2-0.9)	0.5 (0.1-0.8)	P^b^=0.548

*Inflammatory status includes congestion, emphysema, pulmonary bullae with interstitial fibrosis, obstructive pneumonia; a. P-value was calculated by Fisher's exact test; b. P-value was calculated by Wilcox test.

## Abbreviations

AF: allele frequency
CNV: copy number variation
DFS: disease-free survival
MBs: methylation blocks
MD: methylome-based malignancy density
NGS: next-generation sequencing
NR: not reached
NSCLC: non-small-cell lung cancer
PNI: perineural invasion
STAS: spread through air spaces
ROC: receiver operating characteristic
VPI: visceral pleural involvement
