# Supplementary material for: The prognostic value of a Methylome-based Malignancy Density Scoring System to predict recurrence risk in early-stage Lung Adenocarcinoma
Source: Theranostics. 2020 Jun 18;10(17):7635–44. doi: 10.7150/thno.44229 (PMC7359091; doi:10.7150/thno.44229)
Supplement: Supplementary file 1 — Supplementary figures and tables. [file thnov10p7635s1.pdf]

Table S1. The methylation block (MB)-based methylation level and clinical characteristics of patients (See separate Excel file).

Table S2. List of 520-cancer related genes

**Gene**							
ABL1	MAGI2	RPTOR	MED12	GSTT1	CDKN1B	XRCC3	XPO1
ABL2	RET	MAP3K1		H3F3A	CDKN1C	EIF1AX	XRCC2
AKT1	ERCC2	RUNX1	MEF2B	H3F3C	CDKN2A	CYP17A1	STAT5A
AKT2	HLA-A	SDHA	MEN1	HGF	CDKN2B	E2F3	GPR124
AKT3	PGR	SDHB	MET	HIST1H1C	CDKN2C	EGFL7	RECQL4
ALK	ACVR1B	SDHC	MITF	HIST1H2BD	CEBPA	ICOSLG	DNMT1
ALOX12B	RICTOR	MCL1		HIST1H3A	CHD1	MAPK1	ELOC
AMER1	CASP8	SDHD	MLH1	HIST1H3B	CHEK1	RHEB	B2M
APC	HDAC2	SETD2	MLH3	HIST1H3C	CHEK2	CALR	RHOA
AR	FGF23	SF3B1	MPL	HIST1H3D	CIC	CD276	IGF1
ARAF	FGF6	SLX4	MRE11A	HIST1H3E	CREBBP	EPCAM	IRF2
ARID1A	FGF7	SMAD2	MSH2	HIST1H3G	CRKL	FAS	ACVR1
ARID1B	H3F3B	SMAD3	MSH3	HIST1H3H	CRLF2	TGFBR1	EIF4E
ARID2	HIST1H3F	SMAD4	MSH6	INPP4A	RAD50	FGF4	RANBP2
ASXL1	HSD3B1	SMARCA4	MTOR	INSR	PTPRD	FGF19	STAT4
ATM	MYOD1	SMARCB1	MUTYH	MAP3K13	RAD52	FH	FCGR2B
ATR	PHOX2B	SMO	MYC	MST1R	RAF1	LRP1B	HDAC4
ATRX	SOX10	SOCS1	MYCL	NCOR1	PTPN11	FBXW7	PARP1
AURKA	TMEM127	PARP2	MYCN	NEB	KDM6A	FANCC	MALT1
AURKB	BACH1	PIK3CD	MYD88	NOTCH2	CSF1R	YAP1	AXIN2
AXIN1	BBC3	NOTCH4	NBN	NOTCH3	CTCF	ZFHX3	SMARCD1
AXL	CENPA	LATS2	NF1	NPM1	CTNNB1	GPS2	CUL4A
BAP1	CTLA4	PARP3	NF2	NRAS	CUL3	PIK3R3	TRAF7
BARD1	GNA13	ASXL2	NFE2L2	NSD1	DAXX	ANKRD11	CHUK
BCL2	SDHAF2	MDC1	NFKBIA	NTHL1	DDR2	CRBN	SLIT2
BCL2L1	RYBP	MST1	NKX2-1	NTRK1	DICER1	EIF4A2	BCL2L2
BCL6	SH2D1A	FGF12	NOTCH1	NTRK2	DNMT3A	ERCC4	BTG1
BCOR	APCDD1	QKI	SOX2	NTRK3	DOT1L	REL	CXCR4

BLM	IL10	BMPR1A	SOX9	NUP93	HIST1H3I	SHQ1	FOXA1
BRAF	KLF4	BCORL1	SPEN	PAK3	RAD54L	LMO1	HDAC1
BRCA1	PDCD1	PAK1	SPOP	PALB2	HIST1H3J	TET1	HIST2H3C
BRCA2	TIPARP	RPS6KB2	SPTA1	PARK2	HIST2H3D	YES1	HOXB13
BRD4	VTCN1	RPS6KA4	SRC	PAX5	HIST3H3	ZRSR2	ID3
BRIP1	WISP3	FGF14	SRSF2	PBRM1	HNF1A	EED	INHA
BTK	GATA4	PIM1	STAG2	PDGFRA	HNF1B	NRG1	NKX3-1
CARD11	GATA6	SH2B3	STAT3	PDGFRB	HRAS	BCL2L11	PMAIP1
CBFB	GID4	MAPK3	STAT5B	PIK3C2G	KMT2C	FANCI	NCOA3
CBL	PDCD1LG2	PAK7	STK11	PIK3CA	IDH1	EGFR	PNRC1
CHD2	LATS1	FAT3	MGA	PIK3CB	IDH2	EMSY	SOX17
CHD4	KEAP1	FANCE	PRKCI	PIK3CG	IGF1R	EP300	ZBTB2
CSF3R	RAD51B	FGFR2	TOP2A	PIK3R1	IGF2	EPHA3	ZNF703
CUL4B	RARA	MAP2K1	NR4A3	PIK3R2	IKBKE	EPHA5	BCL10
DNMT3B	RAD51	FGFR1	PIK3C2B	PLCG2	IKZF1	EPHA7	DNAJB1
FLCN	PPM1D	PPP6C	SUFU	PMS1	IL7R	EPHB1	FGF10
FLT1	PRSS8	GABRA6	SYK	PMS2	INHBA	ERBB2	LYN
FLT3	RAB35	ZNRF3	TBX3	POLD1	INPP4B	ERBB3	PDK1
FLT4	RIT1	ARID5B	TERC	POLE	IRF4	ERBB4	RAD21
FOXL2	XIAP	HSP90AA1	TERT	POM121L12	IRS1	ERCC1	PDPK1
FOXO1	BCR	CYLD	TET2	PPP2R1A	IRS2	ERG	PLK2
FOXP1	EWSR1	KEL	TGFBR2	PPP2R2A	JAK1	ERRFI1	ERCC3
FUBP1	CCND1	PARP4	TNFAIP3	PRDM1	JAK2	ESR1	ERCC5
FYN	RBM10	MAP2K4	TMPRSS2	PREX2	KMT2A	FANCG	LZTR1
GALNT12	CCND2	ZNF217	TNFRSF14	PRKAR1A	JAK3	EZH2	TACC3
GATA1	CCND3	FRS2	TNFSF11	PRKDC	JUN	FAM175A	MAP3K14
GATA2	CCNE1	BIRC3	TOP1	PTCH1	KDM5A	FAM46C	SUZ12
GATA3	CD274	MAX	TP53	PTEN	KDM5C	FANCA	CTNNA1
GLI1	CD79A	TRAF2	TP63	PTPRS	KMT2D	FANCL	DIS3
GNA11	CD79B	KAT6A	TSC1	PTPRT	KIT	FANCF	RFWD2
GNAQ	CDC73	ARFRP1	TSC2	RASA1	RAC1	FGF3	PIK3C3
GNAS	CDH1	DCUN1D1	TSHR	RUNX1T1	RAD51D	FGFR4	EPHA2
GREM1	CDK12	IFNGR1	U2AF1	SNCAIP	RB1	MAP2K2	PTK2

GRIN2A	CDK4	KLHL6	VEGFA	STK40	RNF43	MDM2	
GRM3	CDK6	NEGR1	VHL	TAF1	KRAS	FAT1	FANCM
GSK3B	CDK8	VEGFB	WRN	TCF3	ROS1	MDM4	
GSTM1	CDKN1A	VEGFC	WT1	TCF7L2	RAD51C	FGFR3	ATF1
TRRAP	KDR	FANCD2	RPA1				

Table S3. The univariate analysis on the correlation of selective methylation sites with DFS (n=39)

Gene	cg Number	Methylated (No. of pts)	Unmethylated (No. of pts)	HR	p-value
HOXA9	cg16104915	11	28	0.81	0.919
C1orf114	cg08104202	2	37	1.09	0.972
TRH	cg22512438	9	30	4.34	0.375
SP9	cg08575330	0	39	1.35	0.961
OTX2	cg10042106	15	24	1.44	0.854
NPBWR1	cg26205771	5	34	8.8	0.178
ALX1	cg14996220	6	33	3.4	0.433

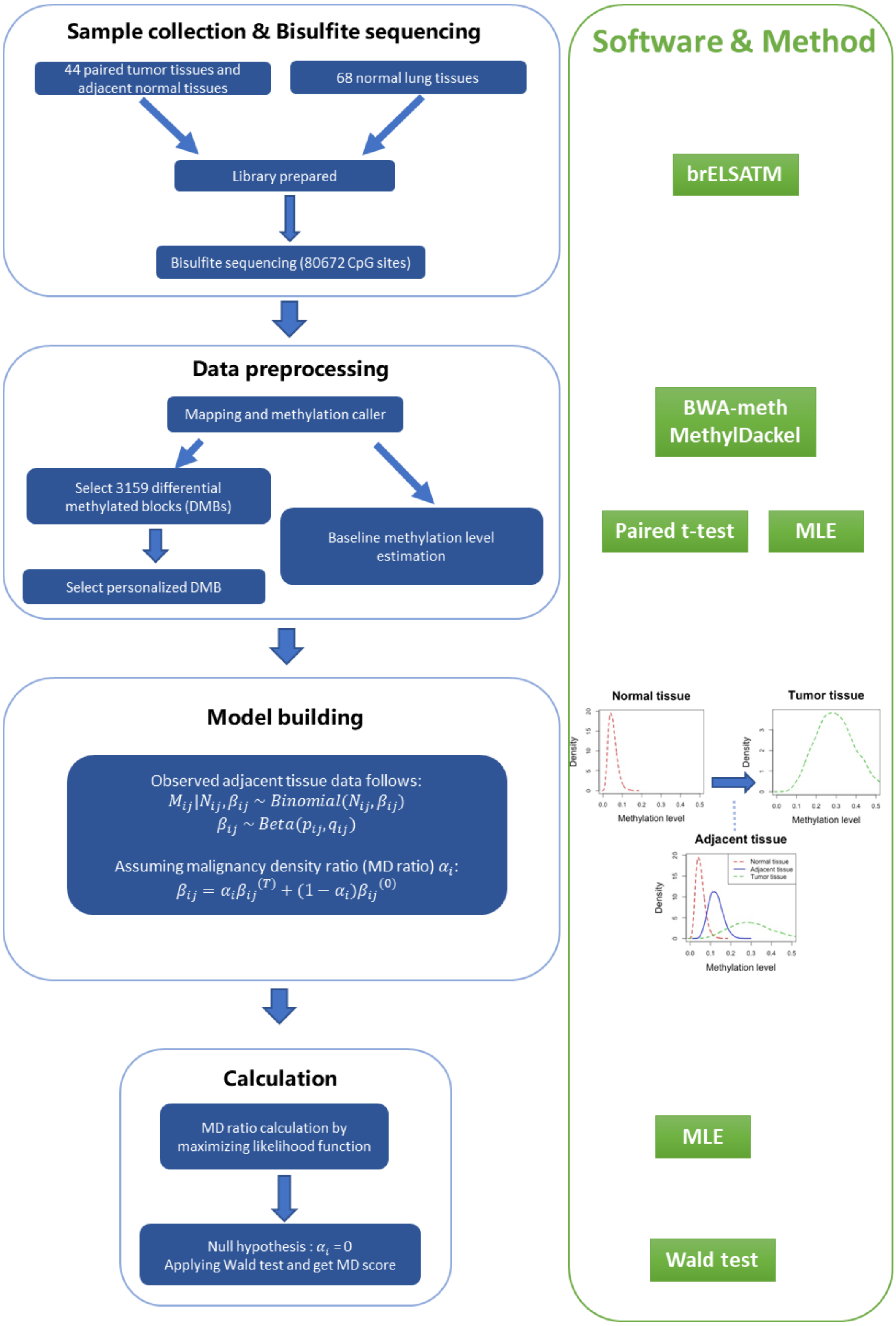

Figure S1. The flowchart of generating MD ratio and MD score.

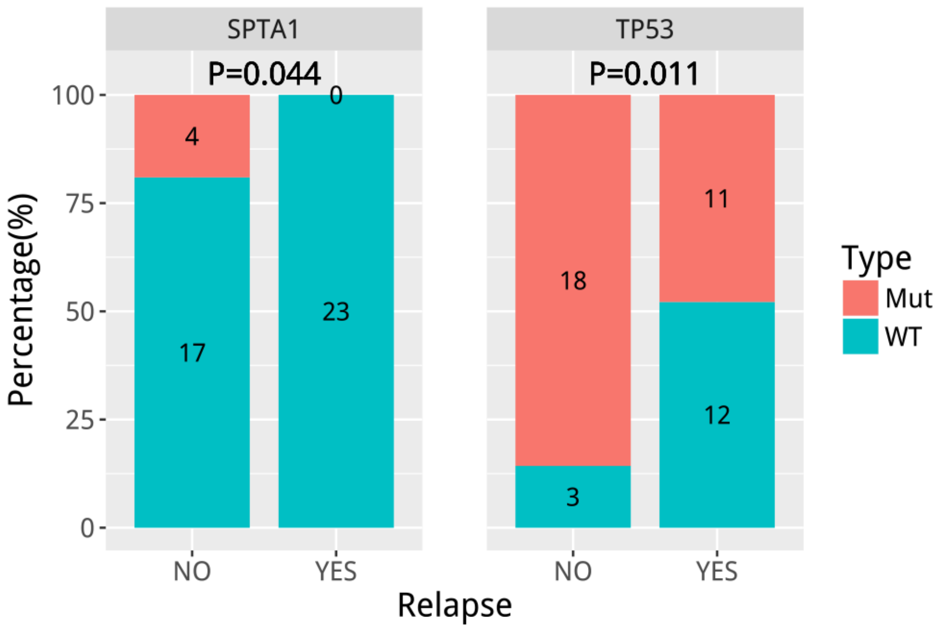

Figure S2. The correlation of genomic alteration with disease relapse.

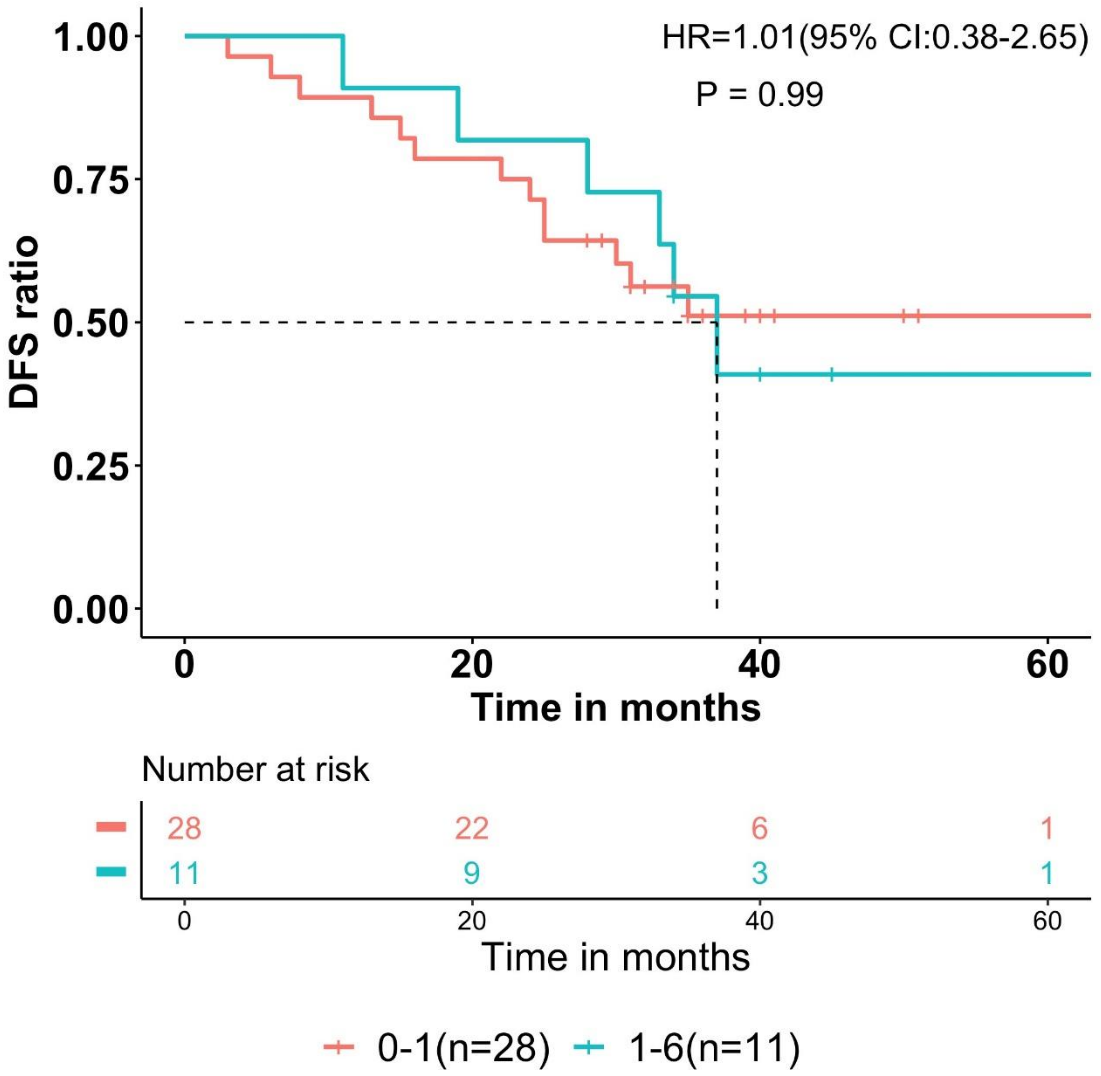

Figure S3. The disease-free survival (DFS) in patients stratified by methylation signature based on 7 selected markers (n=39). Patients were grouped by the number of methylated markers. Markers include *HOXA9, C1orf114, TRH, HIST1H4F, SP9, PCDHGB6, OTX2, NPBWR1, TRIM58, and ALX1*.
